# *FAS-1377 G/A* (rs2234767) Polymorphism and Cancer Susceptibility: A Meta-Analysis of 17,858 Cases and 24,311 Controls

**DOI:** 10.1371/journal.pone.0073700

**Published:** 2013-08-27

**Authors:** Zhou Zhong-Xing, Mi Yuan-Yuan, Ma Hai Zhen, Zou Jian-Gang, Zhang Li-Feng

**Affiliations:** 1 Department of Urology, The Affiliated Changzhou No 2. Hospital of Nanjing Medical University, Changzhou, Jiangsu, China; 2 Department of Urology, The Third Affiliated Hospital of Nantong University, Wuxi, Jiangsu, China; 3 Department of Operating Room, The Third Affiliated Hospital of Nantong University, Wuxi, Jiangsu, China; MOE Key Laboratory of Environment and Health, School of Public Health, Tongji Medical College, Huazhong University of Science and Technology, China

## Abstract

**Background and Objectives:**

Disruption of apoptosis has been implicated in carcinogenesis. Specifically, various single-nucleotide polymorphisms (SNPs) in apoptotic genes, such as *FAS-1377 G/A SNP*, have been associated with cancer risk. *FAS-1377 G/A SNP* has been shown to alter *FAS* gene promoter transcriptional activity. Down-regulation of *FAS* and cell death resistance is key to many cancers, but an association between *FAS-1377 G/A SNP* and cancer risk is uncertain. Therefore, we conducted a meta-analysis of the current literature to clarify this relationship.

**Methodology/Principal Findings:**

From PubMed and Chinese language (CNKI and WanFang) databases, we located articles published up to March 5, 2013, obtaining 44 case-control studies from 41 different articles containing 17,858 cases and 24,311 controls based on search criteria for cancer susceptibility related to the *FAS* gene -1377 G/A *SNP*. Odds ratios (ORs) and 95% confidence intervals (CI) revealed association strengths. Data show that the *-1377 G* allele was protective against cancer risk. Similar associations were detected in “source of control,” ethnicity and cancer type subgroups. Lower cancer risk was found in both smokers with a GG+GA genotype and in non-smokers with the GG+GA genotype, when compared to smokers and nonsmokers with the AA genotype. Males carrying the -1377G allele (GG+GA) had lower cancer incidence than those with the AA genotype. Individuals who carried both *FAS*-1377(*GG*+*GA*)/FASL-844(*TT*+*TC*) genotypes appeared to have lower risk of cancer than those who carried both *FAS-1377 AA/FASL-844 CC* genotypes.

**Conclusions/Significance:**

The *FAS-1377 G/A SNP* may decrease cancer risk. Studies with larger samples to study gene-environment interactions are warranted to understand the role of *FAS* gene polymorphisms, especially -1377 G/A *SNP*, in cancer risk.

## Introduction

In both economically developed and newly developing countries, cancer remains a significant cause of death [1]. Predisposition to cancer may be conferred by certain genetic polymorphisms that arise from single nucleotide polymorphisms (SNPs) [2]. In fact, numerous genome-wide studies of common cancers suggest a number of loci within the genome that, although they have a low-penetrance, may raise an individual’s susceptibility to cancer [3–5].

Apoptosis, the physiological mechanism of “programmed cell death” is crucial for normal tissue development and homeostasis [6], and aberrant regulation of apoptosis correlates with a variety of human diseases, including some cancers [7,8]. FAS (TNFRSF6/CD95/APO-1), a member of the tumor necrosis factor (TNF) receptor super-family, is a trans-membrane receptor involved in apoptotic signal transmission in many cell types. The apoptotic death signal cascade is initiated upon the cross-linking of FAS with its natural ligand (FASL) [9]. Decreased expression or mutation of the FAS gene and/or increased expression of FASL have been reported to occur in many malignant tumors, supposedly impairing the sensitivity of tumor cells to apoptotic signals. Then, tumor cells can evade or weaken the immune system’s ability to eliminate them through the FAS-FASL pathway [10–12]. This may explain correlations between FAS and FASL and human carcinogenesis and/or aggressive tumor behavior [10,11]. Also, decreased FAS expression may protect transformed cells from being eliminated by anti-tumor immune responses, whereas heightened FASL expression may increase the ability of tumor cells to counter-attack the immune system by killing FAS-sensitive lymphocytes, contributing to cancer development [13]. Thus, cancers are not only associated with unlimited cell proliferation, but also with suppression of apoptosis.

**Figure 1:**
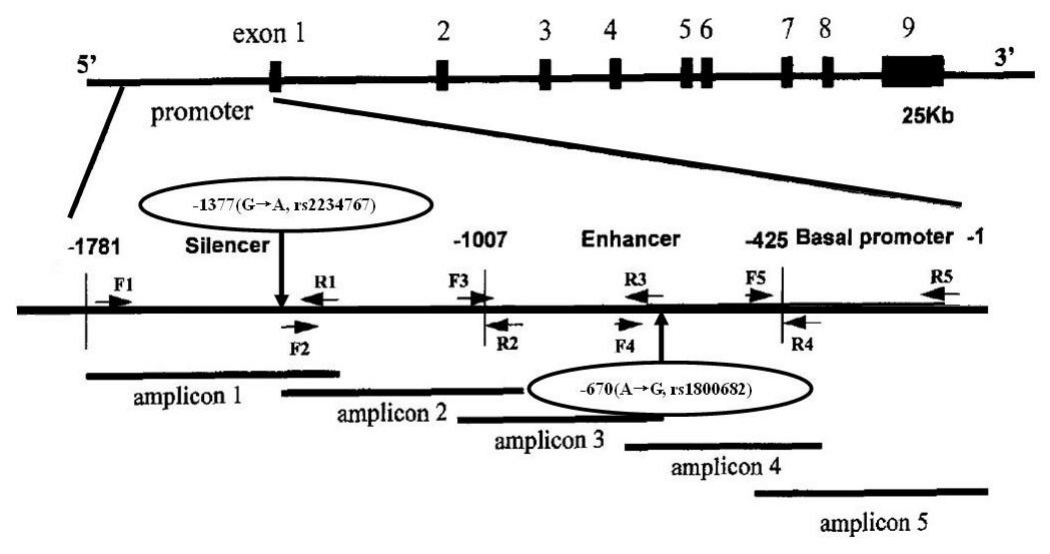
Genomic structure of the human FAS (TNFRSF6/CD95/APO-1) gene with a schematic representation of primer design used to amplify the 5’ flanking region. Five sets of primers were synthesized, ranging from 240–450 bp. The G-to-A substitution polymorphism is located at the -1377 nucleotide position within the silencer region and is situated at the consensus sequence of the transcription factor SP-1 binding site. Another A-to-G substitution polymorphism is located at the -670 position of the promoter region and situated at the binding site of the signal transducer and activator of transcription (STAT) factor. F: forward primer; R: reverse primer. Solid lines represent PCR products, labeled as amplicon 1–5, respectively. Shadowed boxes are exons [15].

The *FAS* gene (GenBank no. AY450925) is located on chromosome 10q24.1, and a polymorphism identified in the FAS promoter region is a G-to-A transition at position -1377 (*FAS-1377 G/A*, rs2234767) [14,15] (Figure 1). This polymorphism destroys the stimulatory protein (Sp) 1 and the signal transducer and activator of transcription (STAT) 1 protein-binding element, diminishing promoter activity and decreasing FAS expression [16,17]. Thus, the G-allele may protect transformed cells against apoptosis, whereas the A-allele maybe a risk factor for cancer.

Many epidemiologic studies suggest associations between SNPs in *FAS* genes, mostly the *FAS-1377 G/A SNP*, and cancer risk. However, conclusions across studies are inconsistent due, in part, to different study populations, case ascertainment, and/or small sample sizes. Thus, previous studies may have identified false-positives as well as suffered from a limited power to detect modest associations. Positive findings were detected in two previously published meta-analyses [18,19], but these studies were not sufficiently large for a comprehensive analysis.

Considering the important role of *FAS-1377 G/A SNP* in carcinogenesis, we studied all currently eligible case-control studies that included characteristics such as ethnicity, cancer type, smoking behaviors, sex, and control sources. Through a meta-analysis of these recent publications, we identified several novel data points, and to our knowledge, ours is the most comprehensive meta-analysis in the literature to study the association between the *FAS-1377 G/A SNP* and cancer risk.

## Materials and Methods

### Identification and eligibility of relevant studies

Searches were conducted in PubMed and in Chinese language (CNKI and WanFang) databases using key words ‘FAS’, ‘cancer’, or ‘polymorphism’. No restrictions were placed on language or publication year and the last search was updated on March 5, 2013. A total of 173 articles were retrieved using the abovementioned terms and 41 articles contained the inclusion criteria. References of the retrieved and review articles were also screened by hand.

### Inclusion criteria and exclusion criteria

Studies that were included in our analysis had to meet all of the following criteria: (1) the study assessed the correlation between cancer risk and the *FAS-1377 G/A SNP*; (2) the study was case-controlled and (3) the study contained sufficient genotype numbers for cases and controls. The following exclusion criteria were used: (1) lack of a control population; (2) lack of available genotype frequency data; and (3) the study was a duplicate.

### Data extraction

Two of the authors extracted all data independently according to the selection criteria. The following items were collected: last name of first author, year of publication, country of origin, ethnicity, cancer type, the total and number of each genotype frequency in case/control groups, ‘source of control’, Hardy-Weinberg equilibrium (HWE) of controls, and genotyping methods. Subgroup analysis, stratified by cancer type, was performed. If a cancer type appeared in only one study, it was placed into the ‘other cancers’ subgroup. Ethnicity was categorized as Caucasian and Asian. The ‘source of control’ subgroup analysis was performed on two groups and was classified as population-based (PB) or hospital-based (HB). Smoking (smoker or non-smoker) status and subject sex (man or woman) were also included in our meta-analysis.

### Statistical analysis

Crude odds ratios (ORs) with 95% confidence intervals (CI) were used to measure the strength of the association between the *FAS-1377 G/A SNP* and cancer risk based on genotype frequencies in cases and controls. The fixed-effects model and the random-effects model were used to calculate the pooled OR value. The statistical significance of the summary OR was determined with the *Z*-test. A heterogeneity assumption was evaluated among studies using a Chi-square-based *Q* test. A *P* value of more than 0.10 for the *Q*-test indicated a lack of heterogeneity among the studies. If significant heterogeneity was detected, the random-effects model (DerSimonian-Laird method) was used. Otherwise, the fixed-effects model (Mantel-Haenszel method) was chosen [20,21].

We investigated the relationship between genetic variants of the *FAS-1377* site and cancer risk by allelic contrast (G-allele vs. A-allele), comparison of homozygotes (GG vs. AA), comparison of heterozygotes (GA vs. AA) and the dominant genetic model (GG+GA vs. AA). Sensitivity analysis was performed by assessing the stability of the results after omitting each study, one at a time. The departure of the *FAS-1377 G/A SNP* from expected frequencies under HWE was assessed in controls using the Pearson Chi-square test (*P* < 0.05 was considered significant). Moreover, the multiplicative gene-gene interactions between *FAS-1377G>A* and *FASL-844T>C* polymorphisms was tested. Publication bias was identified using Egger’s linear regression method and a funnel plot. A *P*-value < 0.05 in Egger’s linear regression indicated the presence of potential publication bias [22]. All statistical tests for this meta-analysis were performed with STATA software (version 10.0; StataCorp LP, College Station, TX).

### Genotyping methods

Methods for genotyping for the *FAS* gene -1377 G/A *SNP* was conducted in the retrieved literature using the polymerase chain reaction-restriction fragment length polymorphism (PCR-RFLP), the ligase detection reaction-polymerase chain reaction (LDR-PCR), and Taqman technology.

## Results

### Study characteristics

**Figure 2:**
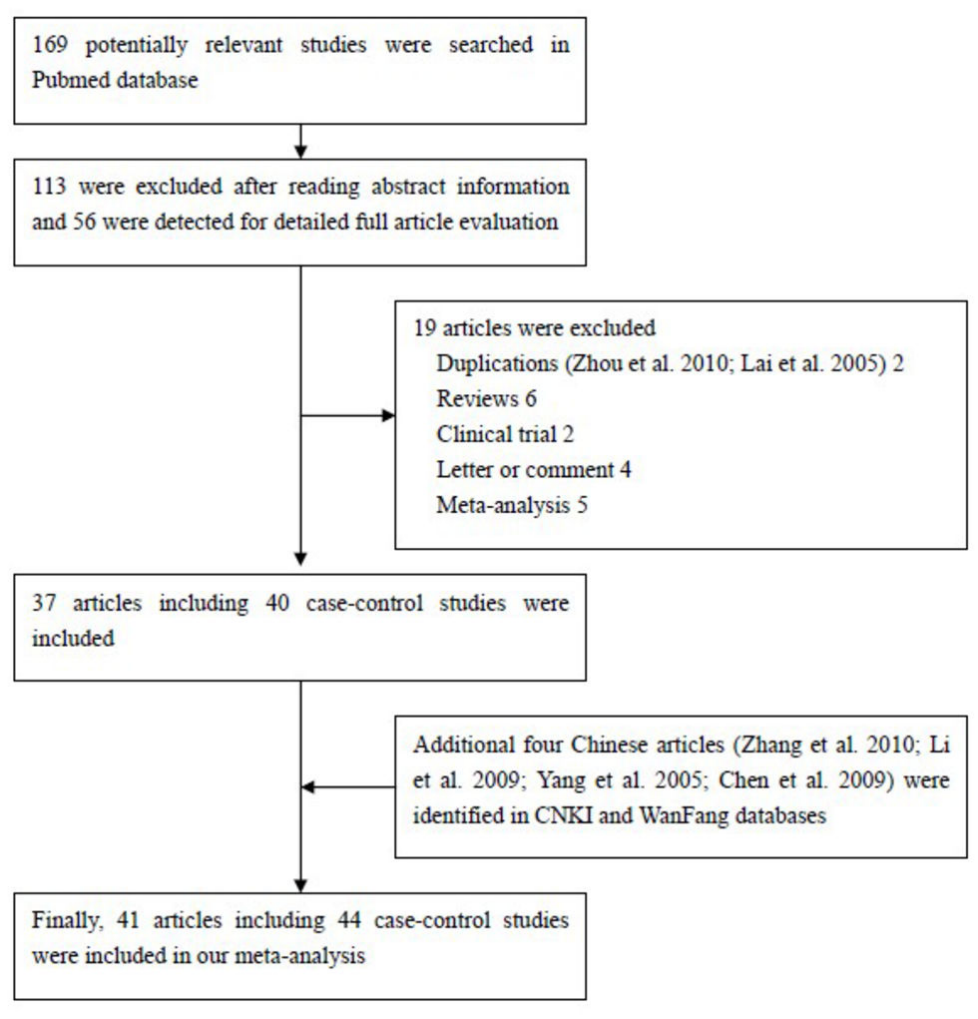
Flowchart illustrating the search strategy used to identify association studies of FAS-1377 G/A polymorphisms and overall cancer risk for the meta-analysis. A total of 173 published studies assessing the association of *FAS-1377 G/A* polymorphisms and cancer were identified by searching the Pubmed and WanFang databases. Through abstract appraisal, 59 articles were identified as eligible for full-text appraisal. From these, an additional 19 articles (2 duplications, 6 reviews, 2 clinical trials, 4 letters/comments and 5 meta-analyses) were excluded. Finally, 41 articles involving 44 case-control design, and data from these were extracted for further assessment in the meta-analysis.

**Figure 3:**
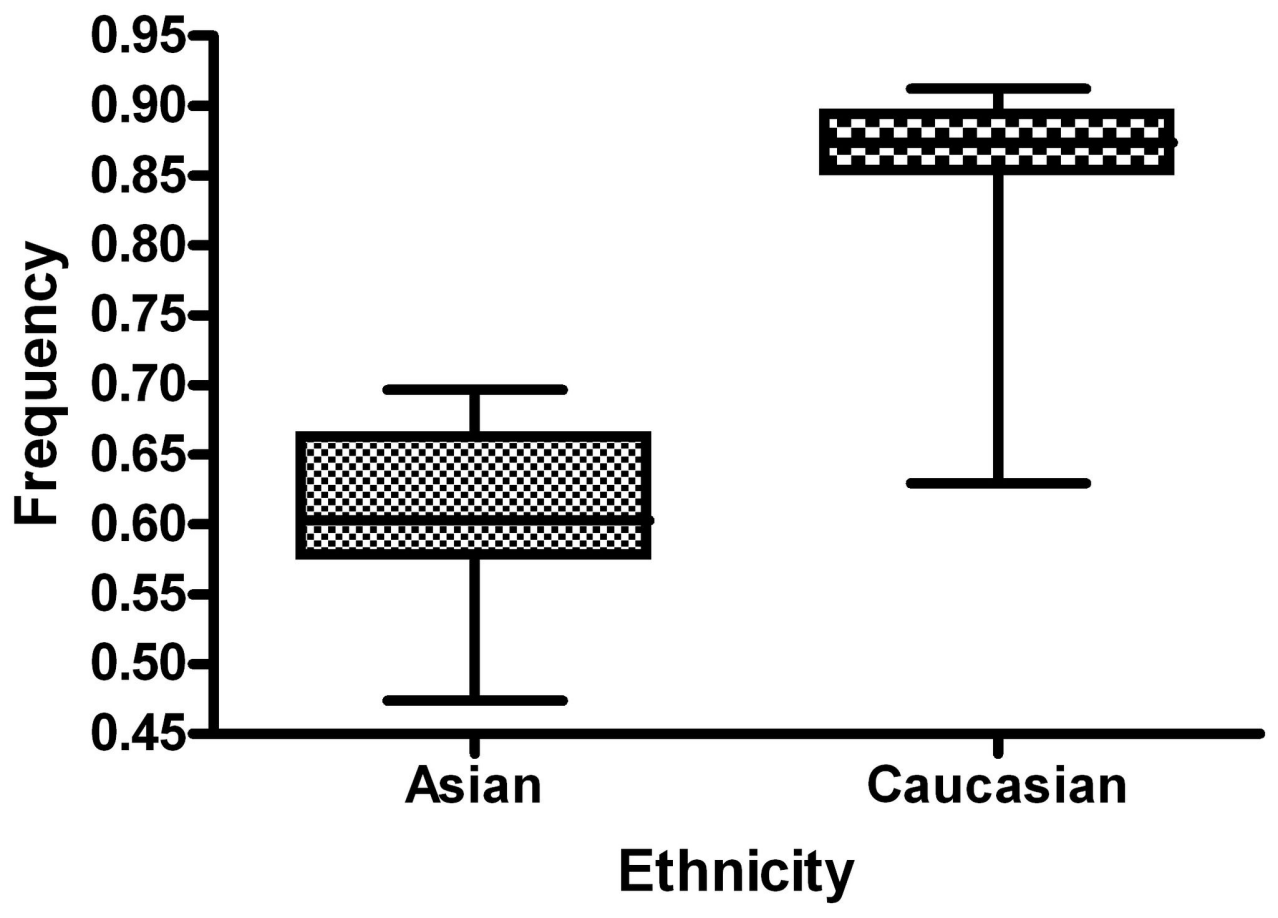
G allele frequencies of *FAS-1377* G/A polymorphism among cases stratified by ethnicity. The *-1377* G-allele frequency is 0.612 in Asian populations and 0.855 in Caucasians. The G-allele frequency in Asian cases was lower than that in European cases (*P* < 0.001). Vertical line: G-allele frequency; Horizontal line: ethnicity type.

**Figure 4:**
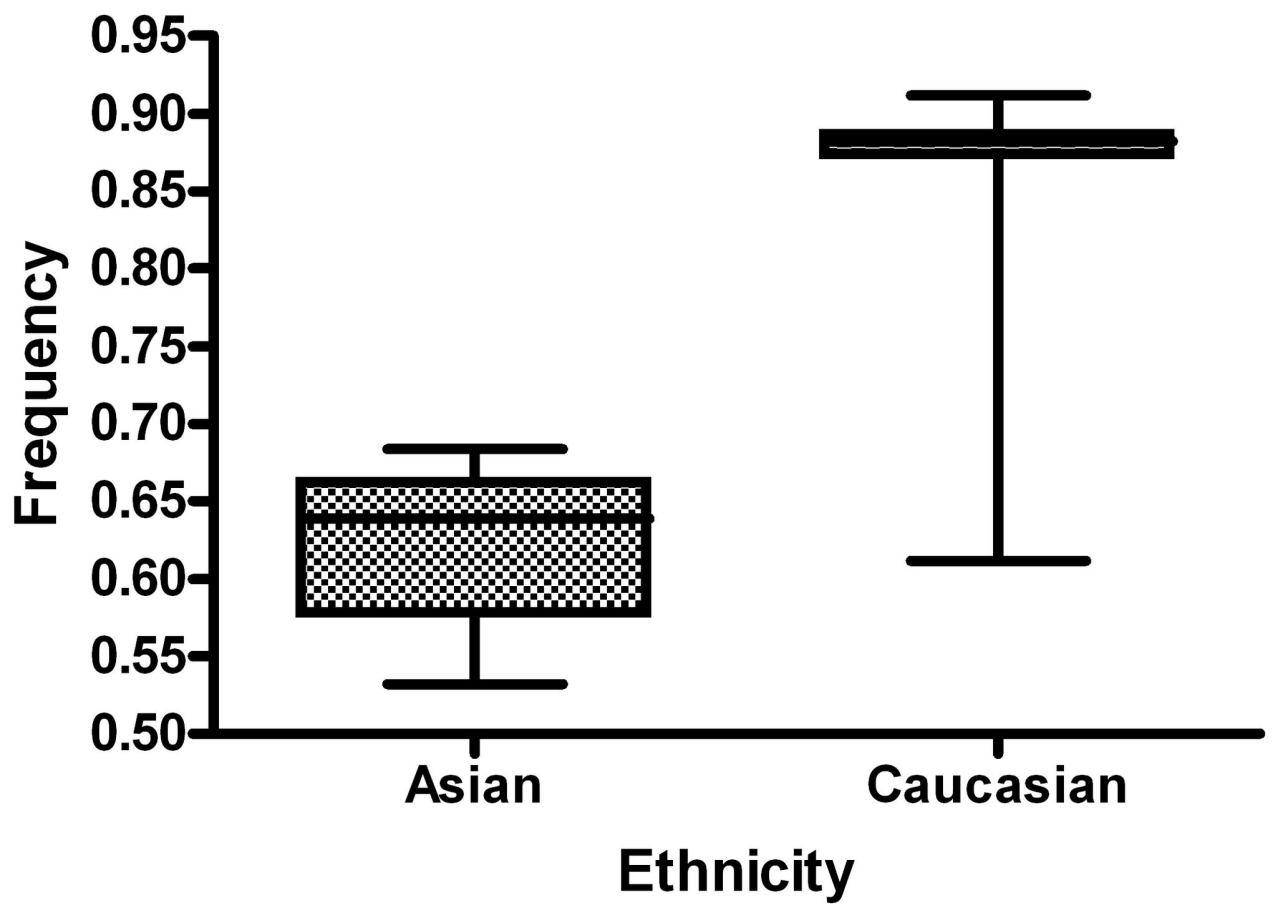
G allele frequencies of *FAS-1377* G/A polymorphism among controls stratified by ethnicity. The *-1377* G-allele frequency is 0.623 in Asian populations and 0.862 in Caucasians. The G-allele frequency in Asian cases was lower than in European cases (*P* < 0.001). Vertical line: G-allele frequency; Horizontal line: ethnicity type.

A total of 169 articles were collected from the PubMed and Chinese language (CNKI and WanFang) databases via a literature search using different combinations of key terms. As shown in Figure 2, 41 articles (44 case-controlled studies including 17,858 cases and 24,311 controls) were ultimately identified [23–63]. Study characteristics from published studies on the relationship between *FAS-1377 G/A SNP* and cancer risk are summarized in Table S1. The frequency of the G-allele was found to be significantly lower in control individuals of Asian ethnicity than in those of Caucasian ethnicity (*P*<0.001). A similar trend was found for the G-allele among Asian and Caucasian individuals with cancer (Figures 3 and 4). The distribution of genotypes among controls was consistent with HWE in all studies except six [37,45,51,53,57,62]. Seven different articles included the genotype detail and smoking status, and three articles included information regarding sex. In most of the studies, cases were histologically diagnosed, and controls were cancer-free. Six publications [25,28,45,46,52,55] contained information about gene-gene interactions between *FAS-1377G/A* and *FASL-844T/C* polymorphisms.

### Quantitative synthesis

The results of the overall meta-analysis suggested a decreased association between the *FAS-1377G/A SNP* and cancer susceptibility (Homozygote comparison: OR = 0.86, 95% CI = 0.78-0.96, *P*_heterogeneity_ = 0.004, *P* = 0.006, dominant model: OR = 0.85, 95% CI = 0.78-0.94, *P*_heterogeneity_ = 0.010, *P* = 0.001 and allelic contrast: OR = 0.95, 95% CI = 0.91-1.00, *P*_heterogeneity_ = 0.000, *P* = 0.038). The overall association did not change after excluding the five studies that did not agree with HWE (Table 1).

**Table 1:** Total and stratified analysis of Fas -1377G/A SNP on cancer risk.

Variables	N^a^	Cases/Controls	Allelic contrast				Homozygote comparison				Heterozygote comparison				Dominant genetic model		
			OR(95%CI)	*P* ^b^	*P* ^c^		OR(95%CI)	*P* ^b^	*P* ^c^		OR(95%CI)	*P* ^b^	*P* ^c^		OR(95%CI)	*P* ^b^	*P* ^c^
Total	44	17858/24311	0.95(0.91-1.00)	0.000	0.038		0.86(0.78-0.96)	0.004	0.006		1.00(0.94-1.06)	0.015	0.980		0.85(0.78-0.94)	0.010	0.001
HWE	38	15671/21658	0.95(0.90-1.00)	0.000	0.040		0.85(0.77-0.96)	0.005	0.009		0.99(0.93-1.06)	0.006	0.876		0.85(0.77-0.94)	0.019	0.001
Ethnicity									
Asian	29	11059/14201	0.95(0.90-1.01)	0.001	0.086		0.87(0.77-0.98)	0.001	0.020		1.03(0.96-1.09)	0.157	0.419		0.86(0.77-0.95)	0.002	0.004
Caucasian	15	6799/10130	0.94(0.85-1.05)	0.016	0.278		1.00(0.99-1.00)	0.433	0.163		0.99(0.97-1.01)	0.585	0.326		1.00(0.99-1.00)	0.495	0.194
Source of control									
HB	19	5518/7546	0.94(0.87-1.02)	0.016	0.135		0.81(0.67-0.96)	0.019	0.017		1.02(0.99-1.02)	0.203	0.153		0.97(0.96-0.99)	0.103	0.000
PB	25	11623/15945	0.96(0.90-1.02)	0.001	0.157		0.90(0.79-1.03)	0.031	0.113		0.91(0.80-1.03)	0.055	0.122		0.90(0.79-1.02)	0.022	0.114
Cancer type									
Gastric cancer	7	1747/2328	0.98(0.95-1.01)	0.300	0.128		0.95(0.91-0.99)	0.404	0.030		1.01(0.95-1.08)	0.876	0.720		0.97(0.95-0.99)	0.400	0.015
Prostate cancer	2	794/927	1.05(1.00-1.11)	0.138	0.063		1.06(0.97-1.06)	0.297	0.179		1.00(0.94-1.07)	0.771	0.962		1.01(0.97-1.05)	0.753	0.486
Leukemia	3	1424/2308	0.94(0.65-1.36)	0.000	0.745		1.01(0.62-1.64)	0.029	0.975		1.02(0.97-1.07)	0.671	0.525		1.01(0.99-1.03)	0.133	0.840
Cervical cancer	4	1100/1706	1.01(0.97-1.05)	0.334	0.489		1.01(0.96-1.07)	0.362	0.629		0.98(0.93-1.03)	0.224	0.477		1.00(0.97-1.02)	0.306	0.815
Esophageal carcinoma	2	776/972	0.97(0.93-1.02)	0.082	0.319		0.84(0.41-1.77)	0.038	0.658		0.73(0.33-1.61)	0.026	0.433		0.79(0.37-1.70)	0.023	0.544
Lung cancer	4	3806/3443	0.99(0.97-1.01)	0.177	0.224		0.85(0.59-1.22)	0.050	0.377		0.79(0.56-1.12)	0.064	0.184		0.82(0.58-1.16)	0.044	0.255
Ovarian carcinoma	2	389/385	1.00(0.94-1.07)	0.298	0.963		-		-		-
Melanoma	3	1039/1789	1.01(0.99-1.04)	0.170	0.182		1.01(1.00-1.02)	0.743	0.208		1.02(0.97-1.07)	0.437	0.427		1.01(1.00-1.02)	0.693	0.239
Skin carcinoma	2	570/1670	0.97(0.95-1.00)	0.322	0.036		0.99(0.97-1.01)	0.302	0.202		0.98(0.93-1.03)	0.447	0.437		0.99(0.98-1.01)	0.329	0.238
Breast cancer	4	2406/2593	0.97(0.95-0.99)	0.315	0.039		0.96(0.94-0.99)	0.339	0.005		0.96(0.92-1.01)	0.118	0.110		0.98(0.96-1.00)	0.173	0.025
Other cancers	11	3807/6210	0.97(0.95-0.99)	0.011	0.002		0.96(0.94-0.98)	0.000	0.000		0.95(0.92-0.98)	0.119	0.002		0.97(0.96-0.99)	0.204	0.000
Smoking status									
Smoker	7	1968/1993	-				-				-				0.92(0.90-0.95)	0.104	0.000
Non-smoker	6	1175/1974	-				-				-				0.95(0.92-0.98)	0.073	0.004
Sexual status									
Man	3	908/1074	-				-				-				0.93(0.89-0.96)	0.230	0.000
Women	2	168/221	-				-				-				0.95(0.88-1.03)	0.360	0.205

^a^ Number of comparisons, ^b^ *P* value of Q-test for heterogeneity test, ^c^ *P*-value of *Z*-test for significant test

In the stratified analysis by cancer type, a significant association was identified between the *FAS-1377G/A SNP* and gastric cancer, skin cancer, breast cancer and other cancers (gastric cancer: OR = 0.95, 95% CI = 0.91-0.99, *P*_heterogeneity_ = 0.404, *P* = 0.030 for GG vs. AA and OR = 0.97, 95% CI = 0.95-0.99, *P*_heterogeneity_ = 0.400, *P* = 0.015 for GG+GA vs. AA; skin cancer: OR = 0.97, 95% CI = 0.95-1.00, *P*_heterogeneity_ = 0.322, *P* = 0.036 for the G-allele vs. A-allele; breast cancer: OR = 0.97, 95% CI = 0.95-0.99, *P*_heterogeneity_ = 0.315, *P* = 0.039 for the G-allele vs. A-allele, OR = 0.96, 95% CI = 0.94-0.99, *P*_heterogeneity_ = 0.339, *P* = 0.005 for GG vs. AA, OR = 0.98, 95% CI = 0.96-1.00, *P*_heterogeneity_ = 0.173, *P* = 0.025 for GG+GA vs. AA; Other cancers: in all four genetic models). Similarly, a significantly decreased association was found in the HB subgroup (Table 1).

When studies were stratified according to ethnicity, there was a significantly decreased association between the *FAS-1377G/A* SNP and cancer susceptibility in Asians (OR = 0.87, 95% CI = 0.77-0.98, *P*_heterogeneity_ = 0.001, *P* = 0.020 for GG vs. AA and OR = 0.86, 95% CI = 0.77-0.95, *P*_heterogeneity_ = 0.002, *P* = 0.004 for GG+GA vs. AA) (Table 1).

Interestingly, compared to AA genotypes, individuals with GG+GA genotypes had lower cancer risk if they were also smokers (GG+GA vs. AA: OR = 0.92, 95% CI = 0.90-0.95, *P*_heterogeneity_ = 0.104, *P* = 0.000) compared to non-smokers (GG+GA vs. AA: OR = 0.95, 95% CI = 0.92-0.98, *P*_heterogeneity_ = 0.073, *P* = 0.004). Men who carried the -1377G allele (GG+GA) also appeared to have a lower incidence of cancer (GG+GA vs. AA: OR = 0.92, 95% CI = 0.90-0.95, *P*_heterogeneity_ = 0.230, *P* = 0.000) than did women who carried the same allele (GG+GA vs. AA: OR = 0.95, 95% CI = 0.88-1.03, *P*_heterogeneity_ = 0.360, *P* = 0.205) (Table 1).

**Figure 5:**
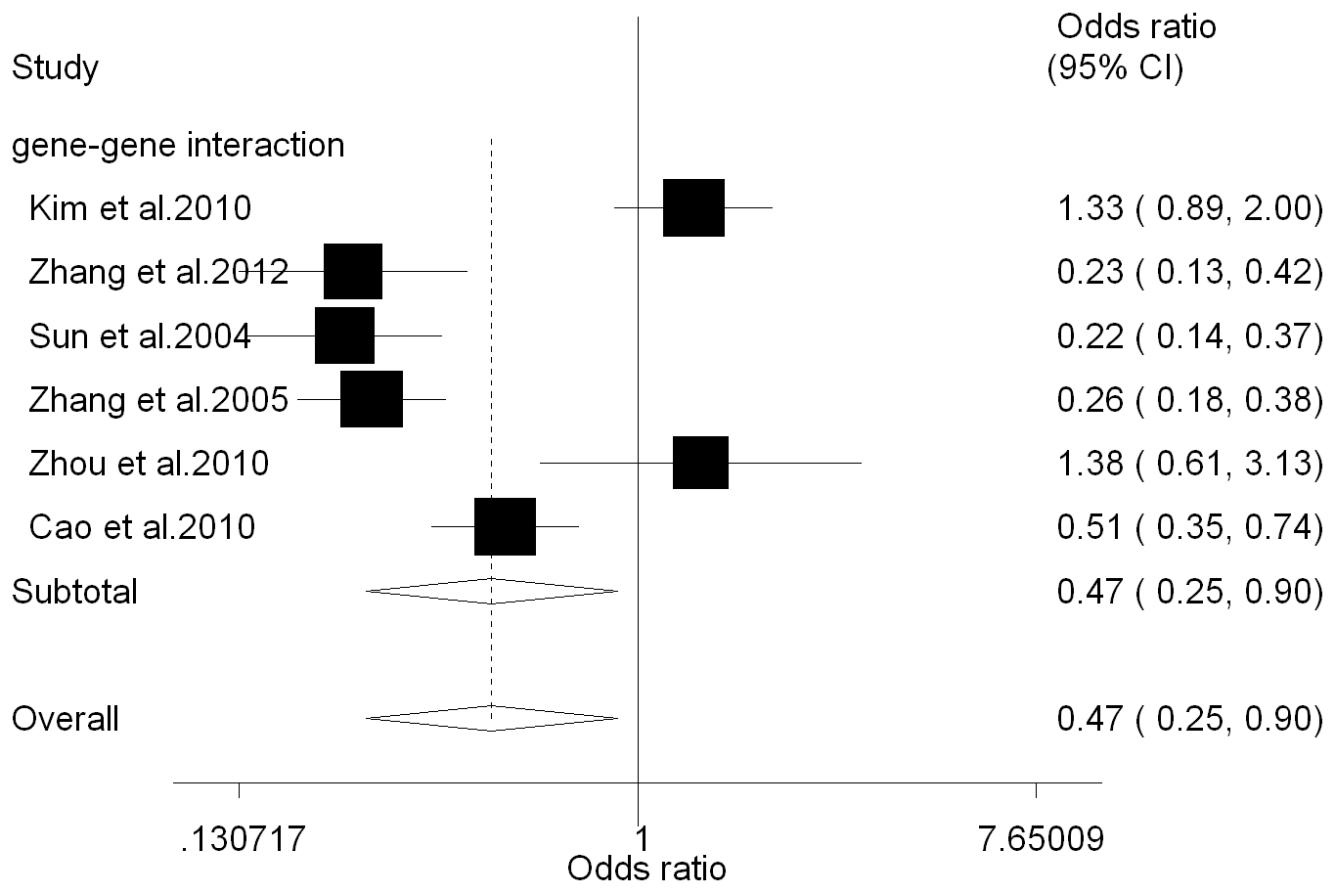
Forest plots illustrate the association of gene-gene interactions between *FAS-1377G/A* and *FASL-844T/C* polymorphisms with cancer risk for GG+GA/TT+TC vs. **AA**/**CC** . For each study, the odds ratio (OR) and 95% confidence interval (CI) values are indicted. The size of each box is proportional to the weight of each study. Diamonds indicate the summary effects based on all studies. The squares and horizontal lines correspond to the OR and 95% CI, and the diamond represents the summary OR and 95% CI.

To evaluate the genotype-genotype interaction, we analyzed the association between cancer risk and the combined genotypes of *FAS-1377G/A* and *FASL-844T/C*. Individuals who carried both *FAS*-1377*(GG*+*GA)*/FASL-844*(TT*+*TC)* genotypes had a decreased cancer risk compared to those who carried both *FAS-1377 AA/FASL-844 CC* genotypes (OR = 0.47, 95% CI = 0.25-0.90, *P*_heterogeneity_ = 0.000, *P* = 0.023) (Table 2, Figure 5). The reduced influence for cancer risk was lower than *FAS* -1377 G/A polymorphism alone.

**Table 2:** Association test for cancer risk with Fas/FasL gene-gene interaction.

Genotypes	Case	Control	OR(95%CI)	P for heterogeneity	*P*	Egger’s test
*FAS -1377(GG+GA)/FASL-844(TT+TC)*	1116	356				
*FAS -1377 AA/FASL-844 CC*	1761	246	0.47(0.25-0.90)	0.000	0.023	T = 0.15, *P* = 0.886

### Sensitivity analysis and bias diagnosis

Using a sensitivity analysis, we investigated whether modification of the inclusion criteria for the meta-analysis affected the final results. No other single study influenced the summary OR qualitatively (data not shown). Egger’s test was performed to assess publication bias and to provide statistical evidence of funnel plot symmetry, and data did not reveal evidence of publication bias.

## Discussion

Studies suggest that down-regulation of the *FAS* gene may protect tumor cells against elimination by anti-tumor immune responses. Furthermore, *FASL* gene up-regulation may increase the ability of tumor cells to counter-attack the immune system via inducing apoptosis of FAS-sensitive lymphocytes [64,65]. Alteration of *FAS* and *FASL* gene expression decreases cellular apoptotic capacities, allowing many tumor cells to evade or suppress the immune system. Most previous studies indicate that decreased *FAS* expression and/or increased *FASL* expression was a common feature of malignant transformation and an early event associated with the development of most human cancers, including gastric cancer, prostate cancer, nasopharyngeal carcinoma, renal cell carcinoma and oral squamous cell carcinoma [25–27,55,66]. Given the critical roles of FAS and FASL in the apoptotic process, it is biologically plausible that an alteration in either of these factors via a genetic polymorphism may affect cancer risk.

To the best of our knowledge, the current report is a timely, updated analysis that combines the findings of all previous publications that evaluated the *FAS-1377G/A SNP* and cancer risk. We performed a meta-analysis involving 17,858 cancer cases and 24,311 healthy controls. In the overall analysis, a decreased association was found between the *FAS-1377G* allele and cancer susceptibility in the three genetic models. Five studies inconsistent with HWE were deleted to increase the power of the current analysis. Similar findings were also indicated for overall cancer risk. In addition, the disruption of the *FAS-1377G/A SNP* diminishes promoter activity and decreases *FAS* gene expression. These findings suggest that the -1377G allele in the *FAS* gene protects against the development of cancer and that the -1377A allele confers an increased risk for the development of cancer.

A vital property of gene polymorphisms is their substantial variation in incidence among different racial or ethnic populations. In the ethnicity subgroup analysis, we found that a significant association between the *FAS-1377G* allele and a decreased risk of cancer in Asians, suggesting genetic-based ethnic diversity. Two possible reasons may explain this difference. On one hand, differences in genetic and environmental backgrounds exist among different ethnicities. On the other hand, different populations usually have different linkage disequilibrium patterns. A polymorphism may be in close linkage with different but nearby causal variants in different populations [67].

In the cancer type subgroup analysis, significant associations were detected between the *FAS-1377 G/A SNP* and skin carcinoma, breast cancer and ‘other cancers’, rather than gastric, lung, and prostate cancers. A possible explanation for this phenomenon is that cancer is a multi-factorial disease that results from complex interactions between many genetic and environmental factors. Thus, a single gene or a single environmental factor is not likely to have a large effect on cancer susceptibility [68].

It is well known that smoking is a risk factor for various diseases, including cancer and that chronic smoking enhances FAS and FASL expression in peripheral blood lymphocytes, which can result in lymphocyte self-destruction or lymphocyte-mediated destruction of other lymphocytes and subsequent immune impairment in smokers [69,70]. The *FAS-1377 SNP* G-to-A substitution destroyed the binding element of transcription factor STAT1, reduced transcription activity, and decreased FAS expression. Possibly, individuals who carry the *FAS-1377 A* allele and smoke may have a higher risk of cancer, and this concept was supported by the data in our meta-analysis.

In the stratified analysis by ‘source of control’ group, moderate strength was observed in HB but not PB controls. This discrepancy may result from a differential influence of selection criteria in different cancers, as well as the weight of each study, which was dictated by sample size in our meta-analysis. HB controls were not strictly healthy individuals, and confounding results may have arisen from the inclusion of controls who were not disease-free, leading to poor statistical representation and publication bias.

An additive gene–gene interaction was observed between *FAS-1377G/A* and *FASL-844T/C* polymorphisms and decreased risk of cancer [71], suggesting that both polymorphisms may be active in the same causal pathway. The statistical interaction between *FAS-1377G/A* and *FASL-844T/C* polymorphisms is biologically plausible because these two molecules comprise a receptor-ligand system, and apoptotic cell death requires both normal FAS and FASL [72]. Therefore, if a cell carries functional polymorphisms in both genes that affect expression, a greater-than-additive effect is to be expected. In cancer development, transformed cells carrying the FASL-844CC genotype that express increased FASL may create an immuno-privileged site by killing cytotoxic immune cells, thereby escaping host immuno-surveillance. In contrast, reduced FAS expression due to the FAS-1377AA genotype may assist the transformed cells in evading FAS- mediated cell death. Thus, subjects carrying both FAS-1377AA and FASL-844CC could be at higher risk for developing cancer than those carrying either FAS-1377AA or FASL-844CC alone [45,73,74]. In other words, individuals carrying both FAS -1377(GG+GA) and FASL -844(TT+TC) genotypes could be at lower risk for developing cancer than those carrying either FAS-1377(GG+GA) alone, which was consistent with our results.

Meta-analysis is an effective method for investigating various clinical questions by summarizing and reviewing published, quantitative studies. Limitations in the present meta-analysis include the suboptimal number of published studies for a comprehensive analysis, especially in terms of linking smoking status, sex and other cancer types. Secondly, gene–gene and gene–environment interactions as well as interactions between different polymorphic loci of the same gene may modulate cancer risk. Thus, these factors should be included in future research and analysis. In addition, our meta-analysis was based on unadjusted estimates. A more precise analysis should be conducted if individual data are available to adjust for other covariates including age, sex, family history, environmental factors, cancer stage, and lifestyle. Finally, controls may not have been truly healthy individuals. In spite of these limitations, there were two advantages to our meta-analysis. First, a substantial number of cases and controls were pooled from different studies, which significantly increased the statistical power of the analysis. Second, the quality of case–control studies included in the current meta-analysis was satisfactory based on our selection criteria.

In summary, in the present meta-analysis, a significantly decreased association was found between *FAS-1377 G/A SNP* and cancer risk. Specifically, the-*1377G allele* was considered to be a protective factor against cancer. Therefore, further large studies, particularly examining gene–gene and gene–environment interactions, are warranted. These future studies could lead to a better and more comprehensive understanding of the association between the *FAS-1377 G/A* polymorphism and development of cancer risk.

## Supporting Information

Table S1 **Study characteristics from published studies on the relationship between Fas -1377 G/A SNP and cancer risk.**
(DOC)
