# Supplementary material for: FAS-1377 G/A (rs2234767) Polymorphism and Cancer Susceptibility: A Meta-Analysis of 17,858 Cases and 24,311 Controls
Source: PLoS One. 2013 Aug 27;8(8):e73700. doi: 10.1371/journal.pone.0073700 (PMC3754923; doi:10.1371/journal.pone.0073700)
Supplement: Table S1 — Study characteristics from published studies on the relationship between Fas -1377 G/A SNP and cancer risk. (DOC) [file pone.0073700.s001.doc]

| First author | Year | Country | Ethnicity | Cancer Type | Source | Case | Control | HWE | Genotype Method |
| --- | --- | --- | --- | --- | --- | --- | --- | --- | --- |
|  |  |  |  |  | of Control | Total(GG/GA/AA) | Total(GG/GA/AA) |  |  |
| Kupcinskas | 2011 | Germany | Caucasian | gastric cancer | HB | 114(95/18/1) | 238(197/40/1) | Yes | TaqMan |
| Shao | 2011 | China | Asian | prostate cancer | HB | 602(253/270/79) | 703(241/359/103) | Yes | PCR-RFLP |
| Cao | 2010 | China | Asian | nasopharyngeal carcinoma | HB | 576(141/264/171) | 608(172/303/133) | Yes | PCR-RFLP |
| Zhu | 2010 | China | Asian | renal cell carcinoma | HB | 353(124/173/56) | 365(161/161/43) | Yes | PCR-RFLP |
| Wang | 2010 | Taiwan | Asian | oral squamous cell carcinoma | PB | 294(95/141/58) | 333(115/165/53) | Yes | PCR-RFLP |
| Zhou | 2010 | China | Asian | gastric cancer | PB | 262(124/117/21) | 524(225/251/48) | Yes | PCR-RFLP |
| Wang | 2009 | China | Asian | gastric cancer | PB | 332(137/156/40) | 324(148/141/35) | Yes | PCR-RFLP |
| Ter-Minassian | 2008 | USA | Caucasian | lung cancer | PB | 2174(1645/492/37) | 1497(1138/336/23) | Yes | TaqMan |
| Yang | 2008 | China | Asian | pancreatic cancer | PB | 397(186/169/42) | 907(420/376/111) | Yes | PCR-RFLP |
| Hsu | 2008 | Taiwan | Asian | gastric cancer | PB | 86(27/42/17) | 101(33/49/19) | Yes | PCR-RFLP |
| Koshkina | 2007 | USA | Caucasian | osteosarcoma | HB | 123(99/22/2) | 510(400/100/10) | Yes | PCR-RFLP |
| Kang | 2008 | Korea | Asian | cervical cancer | HB | 154(54/69/31) | 158(56/82/20) | Yes | PCR-RFLP |
| Crew | 2007 | USA | Caucasian | breast cancer | PB | 1057(809/225/23) | 1106(847/234/25) | Yes | TaqMan |
| Jung | 2007 | Korea | Asian | hepatocellular carcinoma | PB | 312(103/155/54) | 333(119/156/58) | Yes | TaqMan |
| Gormus | 2007 | Turkey | Caucasian | lung cancer | PB | 50(13/37/0) | 94(21/73/0) | No | PCR-RFLP |
| Ho | 2008 | USA | Caucasian | thyroid carcinoma | HB | 279(213/62/4) | 510(400/100/10) | Yes | PCR-RFLP |
| Ho | 2008 | USA | Caucasian | salivary gland carcinoma | HB | 154(126/24/4) | 510(400/100/10) | Yes | PCR-RFLP |
| Gormus | 2007 | Turkey | Caucasian | ovarian carcinoma | HB | 47(28/19/0) | 41(29/12/0) | Yes | PCR-RFLP |
| Zhang | 2007 | China | Asian | breast cancer | PB | 840(293/418/129) | 839(345/382/112) | Yes | PCR-RFLP |
| Zhang | 2006 | USA | Caucasian | head and neck carcinoma | HB | 721(562/142/17) | 1234(957/264/13) | Yes | PCR-RFLP |
| Li | 2006 | USA | Caucasian | melanoma | PB | 602(486/107/9) | 603(459/134/10) | Yes | PCR-RFLP |
| Li | 2006 | China | Asian | bladder cancer | HB | 216(66/104/46) | 252(81/124/47) | Yes | PCR-RFLP |
| Sun | 2005 | China | Asian | cervical cancer | PB | 314(144/144/26) | 615(282/277/56) | Yes | PCR-RFLP |
| Sun | 2005 | China | Asian | esophageal cancer | PB | 588(250/234/104) | 648(273/306/69) | Yes | PCR-RFLP |
| Sibley | 2003 | UK | Caucasian | leukemia | PB | 471(319/136/16) | 931(726/186/19) | Yes | TaqMan/PCR-RFLP |
| Lai | 2007 | Taiwan | Asian | cervical cancer | HB | 318(127/138/53) | 318(99/165/54) | Yes | TaqMan |
| Kim | 2010 | Korea | Asian | leukemia | PB | 592(195/303/94) | 858(286/427/145) | Yes | PCR-RFLP |
| Zhang | 2010 | China | Asian | gastric cancer | HB | 234(99/104/31) | 321(131/151/39) | Yes | PCR-RFLP |
| Li | 2009 | China | Asian | cervical cancer | PB | 314(144/144/26) | 615(282/277/56) | Yes | PCR-RFLP |
| Yang | 2005 | China | Asian | colorectal cancer | PB | 382(152/167/63) | 648(273/306/69) | Yes | PCR-RFLP |
| Chen | 2009 | China | Asian | esophageal cancer | PB | 188(90/80/18) | 324(143/144/37) | Yes | PCR-RFLP |
| Tong | 2012 | China | Asian | leukemia | PB | 361(177/139/45) | 519(212/225/82) | Yes | PCR-RFLP |
| Qureshi | 2010 | USA | Caucasian | melanoma | PB | 208(155/52/1) | 835(657/167/11) | Yes | NA |
| Qureshi | 2010 | USA | Caucasian | skin carcinoma | PB | 274(209/61/4) | 835(657/167/11) | Yes | NA |
| Qureshi | 2010 | USA | Caucasian | skin carcinoma | PB | 296(216/72/8) | 835(657/167/11) | Yes | NA |
| Wang | 2012 | China | Asian | breast cancer | HB | 375(138/171/66) | 496(197/246/53) | Yes | PCR-RFLP |
| Li | 2013 | China | Asian | ovarian carcinoma | PB | 342(159/150/33) | 344(157/151/36) | Yes | LDR-PCR |
| Zhang | 2012 | China | Asian | gastric cancer | HB | 375(138/171/66) | 496(197/246/53) | Yes | PCR-RFLP |
| Mandal | 2012 | India | Asian | prostate cancer | HB | 192(42/116/34) | 224(57/127/40) | No | PCR-RFLP |
| Zhang | 2007 | Sweden | Caucasian | melanoma | PB | 229(183/41/5) | 351(269/70/12) | No | PCR-RFLP |
| Park | 2006 | Korea | Asian | lung cancer | HB | 582(187/300/95) | 582(172/313/97) | No | PCR-RFLP |
| Zhang | 2005 | China | Asian | lung cancer | PB | 1000(413/433/154) | 1270(539/601/130) | No | PCR-RFLP |
| Hashem | 2013 | Iran | Asian | breast cancer | HB | 134(20/10/8) | 152(26/115/11) | No | T-ARMS-PCR |
| Liu | 2011 | China | Asian | gastric cancer | HB | 344(130/155/59) | 324(127/157/40) | Yes | PCR-RFLP |

HB: hospital-based; PB: population-based; PCR-FLIP: polymerase chain reaction and restrictive fragment length polymorphism; LDR-PCR: ligase detection reaction-polymerase chain reaction; NA: not available; T-ARMS-PCR: Tetra-amplification refractory mutation system–polymerase chain reaction
